# Multidrug-Resistant *Salmonella enterica* Serovar Rissen Clusters Detected in Azores Archipelago, Portugal

**DOI:** 10.1155/2019/1860275

**Published:** 2019-12-14

**Authors:** Leonor Silveira, Miguel Pinto, Joana Isidro, Ângela Pista, Patrícia Themudo, Luís Vieira, Jorge Machado, João Paulo Gomes

**Affiliations:** ^1^National Reference Laboratory of Gastrointestinal Infections, Department of Infectious Diseases, National Institute of Health, Avenida Padre Cruz, 1649-016 Lisbon, Portugal; ^2^Bioinformatics Unit, Department of Infectious Diseases, National Institute of Health, Avenida Padre Cruz, 1649-016 Lisbon, Portugal; ^3^National Institute of Agrarian and Veterinary Research, Bacteriology and Micology Laboratory, Avenida da República, Quinta do Marquês, 2780-157 Oeiras, Portugal; ^4^Technology and Innovation Unit, Department of Human Genetics, National Institute of Health, Avenida Padre Cruz, 1649-016 Lisbon, Portugal; ^5^Centre for Toxicogenomics and Human Health (ToxOmics), Genetics, Oncology and Human Toxicology, Nova Medical School/Faculty of Medical Sciences, New University of Lisbon, Avenida Padre Cruz, 1649-016 Lisbon, Portugal

## Abstract

Gastrointestinal infections caused by nontyphoidal *Salmonella* (NTS) remain one of the main causes of foodborne illness worldwide. Within the multiple existing *Salmonella enterica* serovars, the serovar Rissen is rarely reported, particularly as a cause of human salmonellosis. Between 2015 and 2017, the Portuguese National Reference Laboratory of Gastrointestinal Infections observed an increase in the number of clinical cases caused by multidrug-resistant (MDR) *S. enterica* serovar Rissen, particularly from the Azores archipelago. In the present study, we analyzed by whole genome sequencing (WGS) all clinical, animal, food, and environmental isolates received up to 2017 in the Portuguese Reference Laboratories. As such, through a wgMLST-based gene-by-gene analysis, we aimed to identify potential epidemiological clusters linking clinical and samples from multiple sources, while gaining insight into the genetic diversity of *S. enterica* serovar Rissen. We also investigated the genetic basis driving the observed multidrug resistance. By integrating 60 novel genomes with all publicly available serovar Rissen genomes, we observed a low degree of genetic diversity within this serovar. Nevertheless, the majority of Portuguese isolates showed high degree of genetic relatedness and a potential link to pork production. An in-depth analysis of these isolates revealed the existence of two major clusters from the Azores archipelago composed of MDR isolates, most of which were resistant to at least five antimicrobials. Considering the well-known spread of MDR between gastrointestinal bacteria, the identification of MDR circulating clones should constitute an alert to public health authorities. Finally, this study constitutes the starting point for the implementation of the “One Health” approach for *Salmonella* surveillance in Portugal.

## 1. Introduction

Nontyphoidal *Salmonella* (NTS) are zoonotic pathogens that remain one of the main causes of gastrointestinal infection and one of the most important causes of foodborne illness around the world. Annually, an estimated 93.8 million cases of gastroenteritis are caused by NTS worldwide, of which 80.3 million are considered foodborne [1]. Salmonellosis is also estimated to be responsible for 155,000 deaths each year [1]. In 2015, over 95,000 cases of salmonellosis were reported in the European Union (EU) [2]. Although more than 2600 *Salmonella enterica* serovars have been identified to date, most of the cases in developed countries are caused by *S. enterica* serovar Enteritidis or *S. enterica* serovar Typhimurium, accounting for 63% of all reported cases in the EU in 2012 [3, 4]. On the other hand, *S. enterica* serovar Rissen is rarely reported as a cause of human salmonellosis in Europe, but is frequently reported in the United States of America and particularly in Asia [5–7]. As a matter of fact, between 2014 and 2016, this serovar was not even among the 20 most frequently reported serovars responsible for human salmonellosis in the EU/EEA [8]. In Portugal, only 31 cases of human salmonellosis caused by *S. enterica* serovar Rissen were identified in a 12-year period (2000-2012) [9]. However, this is one of the most commonly reported serovars in pigs and pork, in several European and Asian countries [10–17]. This serovar has also been isolated less frequently from other sources, namely, beef, chicken, and seafood [14, 15, 18, 19]. In Portugal, it has been identified in several studies, not only in pig and pork but also in beef, chicken, and wild animals [11, 20–22].

*Salmonella* serotyping has been the gold standard for *Salmonella* surveillance for years, allowing monitoring of shifts in prevalence of certain serotypes in specific regions, which are strong indicatives of existing clusters [23–25]. Until recently, *Salmonella* outbreak investigations have been conducted using different molecular typing methods, such as phage typing, MLVA, or PFGE [26–29]. With the development of next-generation sequencing technologies, those classical typing methods are being used to a lesser extent and genomic approaches based on single nucleotide polymorphisms and wgMLST-based gene-by-gene analysis are progressing as frontline tools for high-resolution isolate characterization and outbreak detection [30–32].

Between 2015 and 2017, an increase in the number of *S. enterica* serovar Rissen isolated from clinical samples, especially multidrug-resistant (MDR) isolates from the Azores archipelago, was observed. We used whole genome sequencing (WGS) to analyze all clinical isolates received from 2014 up to 2017 at the National Reference Laboratory (NRL) of Gastrointestinal Infections at the Portuguese National Institute of Health (INSA), in order to gain insight into the genetic diversity of *S. enterica* serovar Rissen Portuguese (PT) isolates and eventually identify suspected outbreaks. All animal, food, and environmental *S. enterica* serovar Rissen isolates received at the NRL from the National Institute of Agrarian and Veterinary Research (INIAV), between 2014 and 2017, were also included in this work to investigate potential sources of infection.

## 2. Materials and Methods

### 2.1. Bacterial Isolate Typing and Antimicrobial Susceptibility Testing

*S. enterica* isolates included in the present study were obtained from the INSA and INIAV culture collections. The isolates were serotyped by the slide agglutination method, according to the Kauffman-White-Le Minor scheme [23]. In total, 60 *S. enterica* serovar Rissen isolates, collected from 2014 to 2017 in Portugal, were selected for WGS (Supplementary [Supplementary-material supplementary-material-1]). Twenty-two were isolated from human clinical samples, 14 from animals, mostly pigs (*N* = 9) but also bovine (*N* = 4), and chicken (*N* = 1), 22 from food products of animal origin, and 2 from environmental samples.

Antimicrobial Susceptibility Testing was performed by disc diffusion, following the European Committee on Antimicrobial Susceptibility Testing (EUCAST) [33] recommendations, on a panel of 17 antimicrobials: ampicillin (AMP), amoxicillin-clavulanic acid (AMC), cefoxitin, cefotaxime, ceftazidime, ceftriaxone, cefepime, meropenem, pefloxacin (PEF), nalidixic acid (NAL), gentamicin (GEN), azithromycin (AZM), tetracycline (TET), tigecycline, chloramphenicol (CHL), sulfamethoxazole (SMX), and trimethoprim (TMP). Results were interpreted using current epidemiological cut-off values for nalidixic acid, azithromycin, tetracycline, and sulfamethoxazole or the EUCAST breakpoints for the remainder [33–37]. An isolate was classified as MDR when it presented resistance to three or more antimicrobial classes.

### 2.2. Whole Genome Sequencing and Genome Characterization

DNA was extracted from each PT isolate using the NucliSens easyMAG platform (bioMérieux, France) for total nucleic acid extraction according to the manufacturer's instructions. DNA was then subjected to the NexteraXT library preparation protocol (Illumina, USA) prior to paired-end sequencing (2 × 250 bp or 2 × 150 bp) on either a MiSeq or a NextSeq 550 instrument (Illumina, USA) according to the manufacturer's instructions (detailed in Supplementary [Supplementary-material supplementary-material-1]).

All genome sequences were assembled using the INNUca v3.1 pipeline (https://github.com/B-UMMI/INNUca), an integrative bioinformatics pipeline for read quality analysis and *de novo* genome assembly. Read quality analysis and improvement is performed, respectively, using FastQC v0.11.5 (http://www.bioinformatics.babraham.ac.uk/projects/fastqc/) and Trimmomatic v0.36 [38] (with sample-specific read trimming criteria determined automatically based on FasQC report). Genomes are assembled with SPAdes v3.10 (Bankevich et al. [39]) and subsequently polished using Pilon v1.18 [40], with QA/QC statistics (such as depth of coverage and number of contigs) being monitored and reported throughout the analysis. *In silico* MLST prediction is performed using the *mlst* v2.4 software (https://github.com/tseemann/mlst). The full characterization of isolates, including specimen type and source, sampling date, sequence type (ST), final genome assembly sizes, and depth of coverage values, is reported in Supplementary [Supplementary-material supplementary-material-1].

For all isolates, the serotype was predicted *in silico* using SeqSero software [41]. The ResFinder 3.1 web server [42] (https://cge.cbs.dtu.dk/services/ResFinder/) was used to identify acquired antimicrobial resistance genes and/or chromosomal mutations, using a threshold of 80% identity. The predicted results from both platforms were then compared with antimicrobial susceptibility testing results. After genome annotation using Prokka v1.13 [43], metal tolerance was accessed by inspecting the presence of several genes from different metal export systems, such as the copper tolerance genes *pcoABCDRSE*, silver tolerance genes *silCFBAPRSE*, arsenite transmembrane pump genes *arsABCR*, mercury tolerance genes *merACDE*, and tellurite resistance gene *tehAB* [44].

### 2.3. Additional *S. enterica* Serovar Rissen Genome Dataset

For comparative purposes, all *S. enterica* genomes from serovar Rissen identified in the EnteroBase database were downloaded (on November 2018) from the European Nucleotide Archive (ENA) and were assembled as described above using the INNUca pipeline. After postassembly inspection and confirmation of serotype using SeqSero, a total of 270 genomes from strains isolated worldwide, described in Supplementary [Supplementary-material supplementary-material-1].

### 2.4. wgMLST-Based Gene-By-Gene Analysis

A wgMLST-based gene-by-gene analysis was performed by taking advantage of a publicly available panel of 8558 loci [45] derived from the EnteroBase *Salmonella* wgMLST schema [46], curated and prepared using chewBBACA [47], downloaded on August 2018 (10.5281/zenodo.1323684). Allele calling was performed on all genomes using chewBBACA v2.0.11 [47] with default parameters and a publicly available training file for *S. enterica* (https://github.com/mickaelsilva/prodigal_training_files). Exact and inferred matches were used to construct an allelic profile matrix, where other allelic classifications (see https://github.com/B-UMMI/chewBBACA/wiki) were assumed as “missing” loci. Minimum spanning trees (MSTs) were constructed using the goeBURST algorithm [48] implemented in the PHYLOViZ online web-based tool [49], based on 100% shared loci between all isolates (i.e., shared-genome MLST) [50].

An initial MST was constructed enrolling all genomes (i.e., 60 PT plus 270 retrieved from ENA) to integrate all these novel PT genomes within the known *S. enterica* serovar Rissen diversity. Additionally, in order to perform WGS-based epidemiological cluster analysis, a second MST was constructed enrolling only the 60 novel PT genomes. To increase the resolution power for cluster analysis of the PT isolates for both initial MSTs, we took advantage of PHYLOViZ online 2.0 Beta version (http://online2.phyloviz.net/). This platform allows maximization of the shared genome in a dynamic manner, i.e., for each subset of isolates under comparison, the maximum number of shared loci (at 100%) between them is automatically used for subtree construction. All allelic distance thresholds used during cluster investigation were expressed as percentages of allele differences (AD) (i.e., the number of observed allelic differences divided by the total number of shared loci under comparison). Thus, to explore isolate subsets, a conservative step-by-step approach was performed by applying three allelic distance cut-offs of 1, 0.5, and 0.25% to both initial MSTs, based on previously described data for cluster investigation in a wgMLST-based surveillance [51].

## 3. Results

### 3.1. Antimicrobial Susceptibility and Heavy Metal Tolerance

All antimicrobial resistance phenotype and genotype data, including MDR profiles, are presented in Supplementary [Supplementary-material supplementary-material-1]. Although none of the 60 PT isolates are resistant to either meropenem, cefoxitin, cefotaxime, ceftazidime, ceftriaxone, cefepime, or tigecycline, most are resistant to at least one of the remaining antimicrobials tested (i.e., ampicillin, amoxicillin-clavulanic acid, pefloxacin, nalidixic acid, gentamicin, azithromycin, tetracycline, chloramphenicol, sulfamethoxazole, and trimethoprim). Moreover, resistance to more than one antimicrobial was verified in 88.3% of the isolates and 83.3% are MDR. Only one isolate (PT11) is fully susceptible to the antimicrobials tested (1.7%). Sulfamethoxazole resistance is the most common (83.3%), followed by tetracycline (81.7%), trimethoprim (80.0%), ampicillin (73.3%), chloramphenicol (53.3%), and azithromycin (50.0%) resistance. Of note, two distinct food-associated isolates exhibit resistance to quinolones, with PT60 being resistant to both pefloxacin and nalidixic acid while PT44 only to nalidixic acid. Additionally, only one isolate (PT03) reveals intermediate susceptibility to gentamicin (1.7%). None of the isolates presents the genes that confer resistance to colistin (i.e., the *mcr* genes).

Metal resistance-associated genes for copper (*pcoABCDRSE*), arsenic (*arsABCR*), and tellurite (*tehAB*) were observed in all PT isolates analyzed (Supplementary [Supplementary-material supplementary-material-1]). Thirteen isolates (21.7%) presented the mercury resistance-associated genes *merACDE*, which was always collocated with the ampicillin and sulphonamide resistance genes *bla-*TEM-1B and *sul1*, respectively. All these isolates also presented *cmlA1*, conferring resistance to chloramphenicol, and *dfrA1*, conferring resistance to trimethoprim. Fifty-three isolates (88.3%) also present the complete silver tolerance cassette *silCFBAPRSE*, which was located contiguously with the *pco* gene cluster.

### 3.2. Global Genetic Diversity of *S. enterica* Serovar Rissen

**Figure 1:**
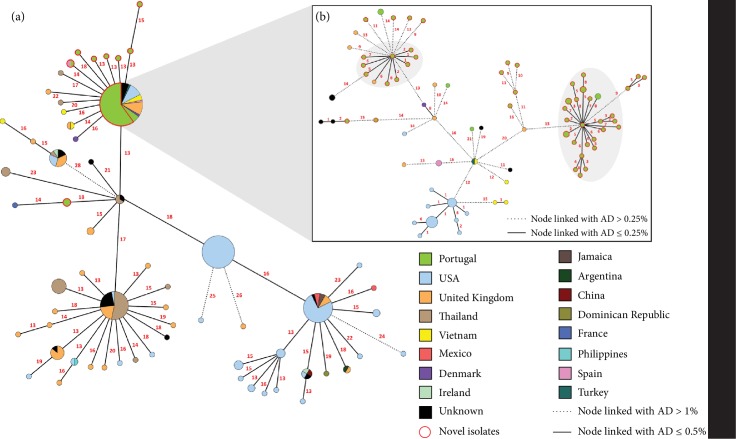
Phylogenetic analysis of *S. enterica* serovar Rissen, based on a gene-by-gene approach using a wgMLST schema with 8558 loci. (a) Minimum spanning tree (MST) enrolling 270 publicly available genomes and the 60 novel “Portuguese” (PT), constructed based on the allelic diversity of 2305 shared loci. The numbers in red on the connecting lines represent the AD between isolates. Nodes linked with allelic distances (AD) equal to or below 0.5% (i.e., 12 AD) have been collapsed for visualization purposes. Node sizes are proportional to the number of isolates they represent. Nodes are colored according to the country of origin. (b) Sub-MST constructed based on the maximum number of shared loci (3162 loci) between the subset of isolates linked at an allelic distance of 0.5% and containing most PT isolates. Two major clusters containing mostly PT isolates linked with AD ≤ 0.25% are highlighted in grey.

All novel PT isolates were firstly integrated with all publicly available *S. enterica* serovar Rissen genomes (*N* = 270), using a wgMLST-based approach, in order to assess their genomic diversity and phylogenetic relationships within the worldwide circulating population. *In silico* seven gene MLST analysis revealed that all enrolled isolates belonged to ST469. The initial MST (Figure 1(a)), based on 2305 shared loci between all 330 isolates, reveals low genetic diversity between all isolates, with an overall mean pairwise AD of 35 ± 9, and that most PT isolates from the present study are closely related. While an initial conservative threshold of 1% (i.e., an AD of 24) still maintains all PT isolates phylogenetically linked, when applying a cut-off of 0.5% (i.e., an AD of 12) to the MST (due to the overall low genetic diversity observed), 10 out of the 60 isolates showed up as unlinked (with two pairs and six single isolates segregating independently) (Figure 1(a)), potentially indicating that they are epidemiologically unrelated. In order to further analyze the cluster containing most PT isolates (at a 0.5% threshold), a sub-MST of this cluster was generated (Figure 1(b)) which increased the number of shared loci to 3162 and an overall mean pairwise AD of 29 ± 10 was observed. This subset of 97 isolates comprises not only most PT isolates but also isolates from the United States of America, the United Kingdom, Spain, Denmark, and Vietnam. Applying a cut-off of 0.5% to this subset, corresponding to an AD of 16, two main clusters containing PT isolates remain and one isolate segregates independently (PT10). Nevertheless, when a more restrict cut-off is applied (0.25%; 8 AD), more consistent with outbreak clustering investigation [51], all the PT isolates separate from strains of other countries (with the exception of an isolate from the United Kingdom, ENA accession # SAMN09298461) and two main clusters containing most of the PT isolates are observed, suggesting two main circulating clones.

### 3.3. WGS-Based Epidemiological Analysis of the PT Isolates

**Figure 2:**
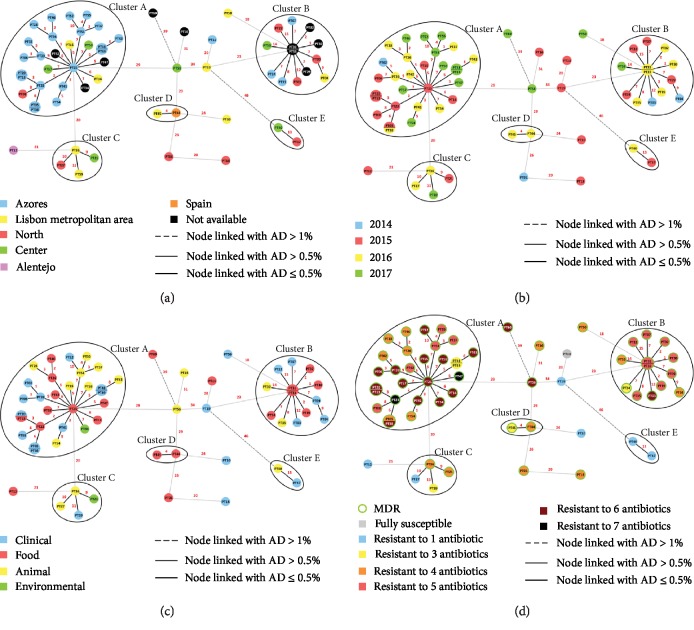
Allelic diversity analysis of the “Portuguese” (PT) *S. enterica* serovar Rissen, using a wgMLST-based gene-by-gene approach. Minimum spanning tree enrolls a total of 3465 shared loci. Nodes are colored by (a) region of isolation, (b) year of isolation, (c) sample type, and (d) antibiotic resistance profile. The numbers in red on the connecting lines represent the allelic distance between isolates. MDR: multidrug resistant.

We then proceeded with the same wgMLST-based approach, strictly for the 60 PT *S. enterica* serovar Rissen isolates, to assess their potential epidemiological relatedness (Figure 2). The initial MST reveals that the isolates share 3465 loci, with a mean pairwise AD of 35 ± 17 (ranging from 0 up to 47). While the number of shared loci between the PT isolates was increased by more than 1100 loci, the overall genetic diversity is still low. As a means to exclude potential epidemiologically unrelated cases of *S. enterica* serovar Rissen within this set of isolates, an initial conservative threshold of 1% (i.e., 35 AD) reveals that at least 3 isolates (PT60, PT40, and PT17) segregate independently. Two linked isolates (cluster E) still sustained their close genetic relatedness (AD of 3) after sub-MST construction (3801 shared loci). Nevertheless, an epidemiological link between these isolates could not be traced. In order to generate potential clusters to be subjected to the dynamic MST analysis, we then applied a lower threshold of 0.5% (i.e., 17 AD) which reveals four additional clusters and nine more potential isolated cases. Cluster D contains two food isolates (Figure 2(c)) retrieved from chicken meat in 2016 (Figure 2(b)), one from the Lisbon metropolitan area (PT45) and the other from Spain (PT44) (Figure 2(a)). After increasing the number of shared loci under analysis to 3828, the sub-MST shows that these isolates are distinguishable by only 7 AD. We also observed a more heterogeneous cluster (Cluster C) where the epidemiological linkage between the four isolates is unclear, due to the differences in all four presented metadata (Figure 2). Still, the sub-MST for this cluster (3805 shared loci) suggests that these isolates are genetically related, as they present a mean pairwise AD of 14 ± 3.

Regarding Cluster B, sub-MST analysis now enrolling 3686 shared loci shows that isolates are still linked at the 0.5% threshold, with a mean pairwise AD of 14 ± 6. Although this cluster is comprised by isolates from animal, food, and clinical samples (Figure 2(c)), it is hard to suggest a direct transmission link from these sources to human, with the clinical cases all detected prior to 2016, contrarily to all but one nonhuman sample (PT24) (Figure 2(b)). However, all isolates from this cluster are MDR (Figure 2(d)). In addition, 12 out of the 13 isolates from this cluster possess the mercury tolerance genes *merACDE*, in association with the chloramphenicol resistance gene, *cmlA1*, and trimethoprim resistance gene *dfrA1* which further distinguishes this cluster from all others where these genes are absent. The only other isolate possessing these genes in the entire dataset is PT50, which is very closely related to this cluster at an AD of 18, suggesting its genetic close relatedness but lacking epidemiological relationship. Moreover, the absence of the silver tolerance-associated genes (*silCFBAPRSE*) was only observed in isolates from this cluster (7 out of the 13, including the five isolates from pork skewers). Of note, the five 2016 isolates from pork skewers with an undisclosed origin (PT29, PT30, PT31, PT32, PT33) are very likely meat products from an identical pig holder, as within 3831 shared loci they only exhibit up to 6 AD between them and share the same resistance profile (AMP-TET-CHL-TMP-SMX). These isolates share the same year of isolation and resistance profile, to both antibiotics and heavy metals, with a pork isolate from the Azores archipelago (PT35) with a maximum AD of 8, all indications of the existence of a possible cluster in Azores.

Finally, the largest cluster (Cluster A) is mostly comprised by isolates from the Azores archipelago (*n* = 21) but also includes two isolates from Lisbon metropolitan area, two from Center region and one from North region (Figure 2(a)). All isolates are still linked after sub-MST construction, sharing 3639 loci with a mean pairwise AD of 12 ± 4. This cluster presents distinct sources (Figure 2(c)), with the majority of isolates (14 out of 29) originating from pigs (Supplementary [Supplementary-material supplementary-material-1]) or being human clinical cases (11 out of 29). Of note, we observed that a clinical isolate (PT20) and a food isolate (PT13), collected two months apart in Azores, presented the same allelic profile, strongly indicating an epidemiological link between them. Moreover, with the exception of PT48 and PT49, all isolates from this cluster are MDR, presenting 4 to 7 resistance determinants (Figure 2(d)). The two non-MDR isolates are likely epidemiologically linked (1 AD between the two) and present the same resistance profile (AMP-AMC-CHL). Most isolates are resistant to azithromycin, with the exception of PT02, PT48, and PT49. In addition, four sets of isolates present the same resistance profiles between them (Supplementary [Supplementary-material supplementary-material-1]): (i) PT26, PT28, and PT46 are TET-TMP-SMX-AZM; (ii) PT37, PT51, and PT55 are AMP-TET-TMP-SMX-AZM; (iii) PT25, PT38, PT39, and PT52 are AMP-AMC-TET-TMP-SMX-AZM; and (iv) PT06, PT13, PT14, PT15, PT20, PT34, PT41, PT43, PT57, and PT58 are AMP-TET-CHL-TMP-SMX-AZM.

## 4. Discussion

WGS is quickly supplanting traditional procedures for *Salmonella* surveillance and outbreak detection in Reference Laboratories. In this regard, food- and water-borne outbreaks are detected either when a common source is determined through epidemiological inquiries, followed by the characterization of all the isolates, or when a group of similar isolates is identified, followed by the common source by epidemiological investigation [30]. The current study aimed for the identification of *S. enterica* serovar Rissen genetic clusters circulating in Portugal, and the detection of potential sources of infection, as a follow-up of an unusual increment in the number of isolates that arrived at the NRLs since 2015.

Even though *S. enterica* serovar Rissen is rarely reported worldwide as a cause of human salmonellosis, it has previously been identified in Portugal associated with pig, pork, beef, chicken, and wild animals [11, 20, 21, 22, 52], which was also observed in this work. Using a dynamic shared-genome-based approach, by progressively maximizing the number of shared loci between isolates, we detected five potential clusters of closely related clinical, animal, food, and environmental *S. enterica* serovar Rissen ST469 isolates [51], with the two largest clusters containing all the isolates from the Azores archipelago (Cluster A and Cluster B) (Figure 2(a)). This approach revealed a high degree of similarity among the *S. enterica* serovar Rissen population, contrary to what was previously described through PFGE [12]. In fact, among the 330 studied isolates, we found a mean genetic distance of about 35 AD (with a maximum AD of 81) within the shared 2305 loci. Apart from a few isolates that segregate independently, a great number of the PT isolates formed very closely related clusters. Increasing the resolution of the initial shared wgMLST approach by increasing the number of loci analyzed reinforced the relatedness of the Portuguese clusters, most specifically the clusters containing MDR isolates from the Azores archipelago (Cluster A and Cluster B). Even though this genomic approach seems to be highly discriminatory, there is no universal cut-off defined for identification of outbreaks; therefore, epidemiological investigation is highly necessary to facilitate the interpretation of WGS data. Given the high degree of genetic similarity within this serovar revealed in this study, several isolates that seem very closely related may in fact be epidemiologically unlinked. Nonetheless, the genomic analysis together with the scarce epidemiological information points to the existence of two nonrelated MDR *S. enterica* serovar Rissen clones circulating in the Azores archipelago for the past years. Additionally, the identification of clinical isolates as well as isolates from animals and food in the Portuguese mainland that show a perfect clustering with the isolates from Azores strongly suggests the spread of the circulating clones throughout the Portuguese territory, with a putative origin in Azores, particularly from pig holding facilities. The fact that the Azores archipelago is composed by nine small islands with livestock as one of the major economic resources reinforces this possibility. Another detected cluster containing a PT isolate and a Spanish isolate (cluster D) seems to suggest the existence of either a *S. enterica* serovar Rissen strain already circulating within the Iberian Peninsula, as a result of intensive trade of live pigs and pork between Portugal and Spain [17], or a discrete phenomenon, as only two cases were detected. Also, these isolates present the resistance genes *sul*1, *dfrA*12, and *aadA*2, mirroring what has been previously reported [17].

Increased antimicrobial resistance in pig-associated *S. enterica* serovars has become a reality for the past decades, including the successful clone *S. enterica* serovar Rissen ST469 [17, 53, 54]. MDR bacteria emerge as a direct consequence of selective pressure derived from overall antibiotic misuse. The use of antibiotics in food-producing animals has been associated with the emergence of certain MDR clones [55]. Additionally, the acquisition of novel properties, such as antibiotic resistance and metal tolerance, may occur by horizontal gene transfer between different bacteria and even between bacterial species [56]. In fact, the success of MDR clones of *S. enterica* serovar Rissen ST469 in pig production has previously been associated with the presence of *pco* and *sil* cassettes [54, 57], as also observed in the present study. Here, 88.3% of the isolates were resistant to more than one antimicrobial and 83.3% were MDR (Supplemental [Supplementary-material supplementary-material-1] and Figure 2(d)). A high level of resistance to several antibiotics was observed, although resistance to carbapenems, cephalosporins, and colistin was not detected. Moreover, 50% of the isolates, mainly isolates from Cluster A, were resistant to azithromycin, which is widely used for the treatment of invasive *Salmonella* infections. According to the genomic analysis of these isolates, azithromycin resistance is likely mediated by the macrolide inactivation gene *mph*A, while *bla*TEM-1B_1 seems to be responsible for ampicillin resistance. Also, *tet*(A) appears in all the tetracycline-resistant isolates of this serovar, confirming that *tet*(A) is most likely the gene responsible for tetracycline resistance in *S. enterica* serovar Rissen [17].

## 5. Conclusions

In summary, we identified at least two MDR *S. enterica* serovar Rissen clones in the Azores archipelago, which are already circulating in Portugal mainland. The presence of MDR isolates with zoonotic potential in food-producing animals is a growing public health concern, having not only a severe burden to human health but also great economic impact. Patients infected by MDR bacteria have an increased risk of developing severe infections with high mortality and morbidity rates, and represent an increased healthcare cost [58]. International trade of food-producing animals and their products contributes greatly to the global spread of MDR *Salmonella* clones, which calls for continuous monitoring, especially in pig production. Although WGS has great potential in supporting epidemiological investigations, the availability of epidemiological data is critical for timely and efficient source detection and outbreak control. This WGS-based *S. enterica* serovar Rissen surveillance study in Portugal results from the collaboration between the Portuguese *Salmonella* NRLs of human and animal health. Hopefully, this stands as the starting point for the implementation of the “One Health” approach for *Salmonella* surveillance in Portugal.

## Data Availability

All raw sequence reads used in the present study were deposited in the ENA under the study accession number PRJEB32515 (individual run accession numbers are detailed in Supplementary [Supplementary-material supplementary-material-1]).

## Supplementary Materials

Supplementary Materials Supplementary Table S1 – Metadata, genome assembly statistics, antibiotic resistance phenotype and genotype and heavy metal tolerance genotype of the Salmonella enterica serovar Rissen isolates enrolled in the present study. Supplementary Table S2 - List of publicly available Salmonella enterica serovar Rissen genomes used in the present study.
