# Supplementary material for: Multidrug-Resistant Salmonella enterica Serovar Rissen Clusters Detected in Azores Archipelago, Portugal
Source: Int J Genomics. 2019 Dec 14;2019:1860275. doi: 10.1155/2019/1860275 (PMC6948285; doi:10.1155/2019/1860275)
Supplement: Supplementary Materials — Supplementary Table S1 – Metadata, genome assembly statistics, antibiotic resistance phenotype and genotype and heavy metal tolerance genotype of the Salmonella enterica serovar Rissen isolates enrolled in the present study. Supplementary Table S2 - List of publicly available Salmonella enterica serovar Rissen genomes used in the present study. [file 1860275.f1.pdf]

Supplementary Table S1A – Metadata, genome assembly statistics, antibiotic resistance phenotype and genotype and heavy metal tolerance genotype of the *Salmonella enterica* serovar Rissen isolates enrolled in the present study.

Isolate ID	Lab. ID	Location	Region (NUTII)	Collection Date	Sample	Source	Product	Mean depth of coverage	Genome size (bp)	# of contigs	ST	Read size (bp)	Sequencing Aparatus	ENA Accession #	ENA ID
PT01	Se_157-14	Braga	North	July 2014	Food	Pig	Fresh sausages	41.14	4907173	108	469	250	MiSeq	ERS3404912	PT_SE0001
PT02	Se_197-14	Azores	Azores	August 2014	Clinical	Human	Urine	64.84	4905194	93	469	150	NextSeq 550	ERS3404913	PT_SE0002
PT03	Se_290-14	Aveiro	North	October 2014	Clinical	Human	Not Available	63.47	4914066	104	469	250	MiSeq	ERS3404914	PT_SE0003
PT04	Se_297-14	Lisbon	Lisbon Metropolitan area	November 2014	Clinical	Human	Faeces	90.53	4919725	112	469	150	NextSeq 550	ERS3404915	PT_SE0004
PT05	Se_4-15	Azores	Azores	January 2015	Clinical	Human	Blood	39.5	4937155	112	469	250	MiSeq	ERS3404916	PT_SE0005
PT06	Se_72-15	Azores	Azores	February 2015	Clinical	Human	Faeces	55.52	4938746	76	469	250	MiSeq	ERS3404917	PT_SE0006
PT07	Se_90-15	Azores	Azores	March 2015	Clinical	Human	Faeces	53.44	4944290	103	469	250	MiSeq	ERS3404918	PT_SE0007
PT08	Se_123-15	Oporto	North	April 2015	Clinical	Human	Faeces	90.93	4892381	107	469	150	NextSeq 550	ERS3404919	PT_SE0008
PT09	Se_128-15	Oporto	North	April 2015	Clinical	Human	Faeces	80.99	4963973	112	469	150	NextSeq 550	ERS3404920	PT_SE0009
PT10	Se_171-15	Lisbon	Lisbon Metropolitan area	June 2015	Clinical	Human	Faeces	58.84	4863424	161	469	150	NextSeq 550	ERS3404921	PT_SE0010
PT11	Se_PIGA-2	Azores	Azores	June 2015	Food	Bovine	Frozen minced meat	53.46	4867520	74	469	250	MiSeq	ERS3404922	PT_SE0011
PT12	Se_PIGA-4	Not Available	Alentejo	June 2015	Food	Not Available	Blood chorizo	56.62	4975525	103	469	250	MiSeq	ERS3404923	PT_SE0012
PT13	Se_PIGA-7	Azores	Azores	June 2015	Food	Pig	Meat chorizo	59.02	4944337	84	469	250	MiSeq	ERS3404924	PT_SE0013
PT14	Se_PIGA-8	Not Available	Lisbon Metropolitan area	June 2015	Food	Pig	Meat	64.89	4945078	102	469	250	MiSeq	ERS3404925	PT_SE0014
PT15	Se_PIGA-9	Not Available	Lisbon Metropolitan area	June 2015	Animal	Pig	Piglet	75.94	4946168	79	469	250	MiSeq	ERS3404926	PT_SE0015
PT16	Se_PVRAM-7-15	Not Available	Not Available	June 2015	Animal	Pig	Piglet carcass	60.58	4995231	144	469	150	NextSeq 550	ERS3404927	PT_SE0016
PT17	Se_227-15	Aveiro	North	July 2015	Clinical	Human	Not Available	52.81	4843182	131	469	150	NextSeq 550	ERS3404928	PT_SE0017
PT18	Se_254-15	Oporto	North	August 2015	Clinical	Human	Faeces	48.15	5012266	89	469	250	MiSeq	ERS3404929	PT_SE0018
PT19	Se_266-15	Setúbal	Lisbon Metropolitan area	August 2015	Clinical	Human	Faeces	63.42	4886200	80	469	250	MiSeq	ERS3404930	PT_SE0019
PT20	Se_270-15	Azores	Azores	August 2015	Clinical	Human	Faeces	42.3	4945266	97	469	250	MiSeq	ERS3404931	PT_SE0020
PT21	Se_305-15	Coimbra	Centre	August 2015	Environmental	NA	Water	125.42	5066848	125	469	250	MiSeq	ERS3404932	PT_SE0021
PT22	Se_479-15	Oporto	North	November 2015	Clinical	Human	Faeces	58.32	4909000	109	469	250	MiSeq	ERS3404933	PT_SE0022
PT23	Se_PIGA-2-16	Azores	Azores	November 2015	Food	Pig	Hamburger	80.69	4940239	84	469	250	MiSeq	ERS3404934	PT_SE0023
PT24	Se_PIGA-3-16	Azores	Azores	November 2015	Food	Pig	Raw Chorizo	48.04	4873930	94	469	250	MiSeq	ERS3404935	PT_SE0024
PT25	Se_PIGA-4-16	Azores	Azores	November 2015	Food	Pig	Raw meat	58.24	4940367	98	469	250	MiSeq	ERS3404936	PT_SE0025
PT26	Se_PIGA-5-16	Azores	Azores	January 2016	Food	Pig	Chorizo	52.54	4938840	85	469	250	MiSeq	ERS3404937	PT_SE0026
PT27	Se_R33-16	Oporto	North	March 2016	Animal	Bovine	Carcass	60.86	4924248	96	469	250	MiSeq	ERS3404938	PT_SE0027
PT28	Se_R39-16	Azores	Azores	March 2016	Animal	Pig	Carcass	55.48	4938922	93	469	250	MiSeq	ERS3404939	PT_SE0028
PT29	Se_R70-16	Not Available	Not Available	May 2016	Food	Pig	Pork skewers	56.54	4879946	112	469	150	NextSeq 550	ERS3404940	PT_SE0029
PT30	Se_R71-16	Not Available	Not Available	May 2016	Food	Pig	Pork skewers	138.65	4880717	91	469	150	NextSeq 550	ERS3404941	PT_SE0030
PT31	Se_R72-16	Not Available	Not Available	May 2016	Food	Pig	Pork skewers	99.05	4889369	102	469	150	NextSeq 550	ERS3404942	PT_SE0031
PT32	Se_R73-16	Not Available	Not Available	May 2016	Food	Pig	Pork skewers	68.58	4876907	110	469	150	NextSeq 550	ERS3404943	PT_SE0032
PT33	Se_R74-16	Not Available	Not Available	May 2016	Food	Pig	Pork skewers	221.84	4881425	87	469	150	NextSeq 550	ERS3404944	PT_SE0033
PT34	Se_R92-16	Not Available	Not Available	May 2016	Environmental	NA	Mud	75.75	4917595	110	469	150	NextSeq 550	ERS3404945	PT_SE0034
PT35	Se_R110-16	Azores	Azores	June 2016	Animal	Pig	Carcass	56.04	4919561	123	469	150	NextSeq 550	ERS3404946	PT_SE0035
PT36	Se_R134-16	Setúbal	Lisbon Metropolitan area	August 2016	Animal	Bivalve	Bivalve mollusc	186.4	4910852	85	469	150	NextSeq 550	ERS3404947	PT_SE0036
PT37	Se_R151-16	Azores	Azores	August 2016	Animal	Pig	Carcass	64.27	4953004	147	469	150	NextSeq 550	ERS3404948	PT_SE0037
PT38	Se_S176	Azores	Azores	August 2016	Clinical	Human	Faeces	71.69	4937185	83	469	250	MiSeq	ERS3404949	PT_SE0038
PT39	Se_S208	Azores	Azores	August 2016	Clinical	Human	Urine	57.31	4941276	117	469	250	MiSeq	ERS3404950	PT_SE0039
PT40	Se_PVRAM-46-16	Viseu	Centre	October 2016	Animal	Chicken	Carcass	68.29	4854659	105	469	150	NextSeq 550	ERS3404951	PT_SE0040
PT41	Se_S308	Azores	Azores	October 2016	Clinical	Human	Exudate	86.48	4979091	118	469	250	MiSeq	ERS3404952	PT_SE0041
PT42	Se_R196-16	Not Available	Not Available	November 2016	Food	Pig	Raw sausage	291.87	4877328	89	469	150	NextSeq 550	ERS3404953	PT_SE0042
PT43	Se_R199-16	Azores	Azores	November 2016	Animal	Bovine	Carcass	39.49	4947413	203	469	150	NextSeq 550	ERS3404954	PT_SE0043
PT44	Se_R200-16	Spain	Spain	November 2016	Food	Chicken	Meat	69.09	4888699	110	469	150	NextSeq 550	ERS3404955	PT_SE0044
PT45	Se_R217-16	Lisbon	Lisbon Metropolitan area	December 2016	Food	Chicken	Raw meat	63.82	4887212	120	469	150	NextSeq 550	ERS3404956	PT_SE0045
PT46	Se_PIGA-7-16	Azores	Azores	January 2016	Food	Pig	Chorizo	77.72	4921583	108	469	150	NextSeq 550	ERS3404957	PT_SE0046
PT47	Se_R65-17	Not Available	Not Available	March 2017	Food	Turkey	Turkey skewers	83.93	4930828	79	469	150	NextSeq 550	ERS3404958	PT_SE0047
PT48	Se_S398	Azores	Azores	March 2017	Clinical	Human	Bronchial aspirate	62.66	4920188	116	469	250	MiSeq	ERS3404959	PT_SE0048
PT49	Se_S402	Azores	Azores	March 2017	Clinical	Human	Urine	46.6	4917517	100	469	250	MiSeq	ERS3404960	PT_SE0049
PT50	Se_S420	Lisbon	Lisbon Metropolitan area	March 2017	Clinical	Human	Urine	39.09	4903866	123	469	250	MiSeq	ERS3404961	PT_SE0050
PT51	Se_R74-17	Azores	Azores	April 2017	Animal	Bovine	Carcass	77.31	4963946	117	469	150	NextSeq 550	ERS3404962	PT_SE0051
PT52	Se_S473	Azores	Azores	May 2017	Clinical	Human	Faeces	46.96	4939749	93	469	250	MiSeq	ERS3404963	PT_SE0052
PT53	Se_PVRAM-2-17	Leiria	Centre	June 2017	Animal	Bovine	Carcass	59.02	4925286	115	469	150	NextSeq 550	ERS3404964	PT_SE0053
PT54	Se_PVRAM-3-17	Azores	Azores	July 2017	Animal	Pig	Carcass	46.47	4936820	229	469	150	NextSeq 550	ERS3404965	PT_SE0054
PT55	Se_PVRAM-10-17	Azores	Azores	August 2017	Animal	Pig	Carcass	127.85	4961638	130	469	150	NextSeq 550	ERS3404966	PT_SE0055
PT56	Se_PVRAM-17-17	Leiria	Centre	August 2017	Animal	Pig	Carcass	49.42	4913428	123	469	150	NextSeq 550	ERS3404967	PT_SE0056
PT57	Se_PIGA-8-17	Not Available	Centre	September 2017	Food	Pig	Chorizo	49.41	4909916	176	469	150	NextSeq 550	ERS3404968	PT_SE0057
PT58	Se_PVRAM-19-17	Leiria	Centre	September 2017	Animal	Pig	Carcass	63.03	4917601	123	469	150	NextSeq 550	ERS3404969	PT_SE0058
PT59	Se_S674	Lisbon	Lisbon Metropolitan area	September 2017	Clinical	Human	Pus	44.34	4924957	182	469	150	NextSeq 550	ERS3404970	PT_SE0059
PT60	Se_R258-17	Not Available	Not Available	October 2017	Food	Bovine/pork	Gourmet Hamburguer	65.2	5090350	122	469	150	NextSeq 550	ERS3404971	PT_SE0060

NUT - Nomenclature of Territorial Units for Statistics.

Supplementary Table S1B – Metadata, genome assembly statistics, antibiotic resistance phenotype and genotype and heavy metal tolerance genotype of the *Salmonella enterica* serovar Rissen isolates enrolled in the present study.

ID	Resistance status	Resistance profile	Beta-lactams					Tetracyclines			Chloramphenicol				Trimethoprim			Sulphonamides			Macrolide		Aminoglycosides						Fluoroquinolones						Heavy metal tolerance				
			AMP	AMC	*bla*TEM-1B_1	CEF*	MEM	TET	TGC	*tet(A)*	CHL	*catA2*	*cmlA1*	*floR*	TMP	*dfrA12*	*dfrA1*	SMX	*sul1*	*sul3*	AZM	*mphA*	GEN	*aac(3)ib*	*aadA2*	*aadA1*	*aac(6')-Iaa*	*aadA24*	PEF	NAL	*qnrS1*	*qnrB14*	*qnrB19*	*parC* (T57S)	*mer ACDE*	*ars ABCR*	*pco ABCDESR*	*pil ABCEFRS*	*tehAB*
PT01	MDR	AMP-TET-TMP-SMX	R	S	+	S	S	R	S	+	S	-	-	-	R	+	-	R	+	-	S	-	S	-	+	+	+	-	S	S	-	-	-	+	-	+	+	+	+
PT02	MDR	TET-CHL-TMP-SMX	S	S	-	S	S	R	S	+	R	+	-	-	R	+	-	R	+	-	S	-	S	-	+	-	+	-	S	S	-	-	-	+	-	+	+	+	+
PT03	MDR	AMP-AMC-TET-CHL-TMP-SMX-GEN	R	R	+	S	S	R	S	+	R	-	+	-	R	-	+	R	+	+	S	-	I	+	+	+	+	-	S	S	-	-	-	+	+	+	+	+	+
PT04	MDR	AMP-CHL-TMP-SMX	R	S	+	S	S	S	S	-	R	-	+	-	R	-	+	R	+	+	S	-	S	-	+	+	+	-	S	S	-	-	-	+	+	+	+	+	+
PT05	MDR	TET-TMP-SMX-AZM	S	S	+	S	S	R	S	+	S	-	-	-	R	+	-	R	+	-	R	+	S	-	-	-	+	+	S	S	-	-	-	+	-	+	+	+	+
PT06	MDR	AMP-TET-CHL-TMP-SMX-AZM	R	S	+	S	S	R	S	+	R	+	-	-	R	+	-	R	+	-	R	+	S	-	+	+	+	-	S	S	-	-	-	+	-	+	+	+	+
PT07	MDR	AMP-TET-CHL-TMP-SMX	R	S	+	S	S	R	S	+	R	-	+	-	R	-	+	R	+	+	S	-	S	-	+	+	+	-	S	S	-	-	-	+	+	+	+	-	+
PT08	MDR	AMP-TET-TMP-SMX-AZM	R	S	+	S	S	R	S	+	S	-	-	-	R	+	-	R	+	-	R	+	S	-	+	+	+	-	S	S	-	-	-	+	-	+	+	+	+
PT09	MDR	AMP-TET-CHL-TMP-SMX	R	S	+	S	S	R	S	+	R	-	+	-	R	-	+	R	+	+	S	-	S	-	+	+	+	-	S	S	-	-	-	+	+	+	+	+	+
PT10	non-MDR	AMP	R	S	+	S	S	S	S	-	S	-	-	-	S	-	-	S	-	-	S	-	S	-	-	-	+	-	S	S	-	-	-	+	-	+	+	+	+
PT11	non-MDR	-	S	S	-	S	S	S	S	-	S	-	-	-	S	-	-	S	-	-	S	-	S	-	-	-	+	-	S	S	-	-	-	+	-	+	+	+	+
PT12	non-MDR	SMX	S	S	+	S	S	S	S	+	S	-	-	-	S	+	-	R	+	-	S	-	S	-	+	+	+	-	S	S	-	-	-	+	-	+	+	+	+
PT13	MDR	AMP-TET-CHL-TMP-SMX-AZM	R	S	+	S	S	R	S	+	R	+	-	-	R	+	-	R	+	-	R	+	S	-	-	-	+	+	S	S	-	-	-	+	-	+	+	+	+
PT14	MDR	AMP-TET-CHL-TMP-SMX-AZM	R	S	+	S	S	R	S	+	R	+	-	-	R	+	-	R	+	-	R	+	S	-	+	+	+	-	S	S	-	-	-	+	-	+	+	+	+
PT15	MDR	AMP-TET-CHL-TMP-SMX-AZM	R	S	+	S	S	R	S	+	R	+	-	-	R	+	-	R	+	-	R	+	S	-	+	+	+	-	S	S	-	-	-	+	-	+	+	+	+
PT16	MDR	TET-TMP-SMX-AZM	S	S	-	S	S	R	S	+	S	-	-	-	R	+	-	R	+	-	R	+	S	-	+	-	+	-	S	S	-	-	-	+	-	+	+	+	+
PT17	non-MDR	TET	S	S	-	S	S	R	S	+	S	-	-	-	S	-	-	S	-	-	S	-	S	-	-	-	+	-	S	S	-	-	-	+	-	+	+	+	+
PT18	MDR	AMP-TET-TMP-SMX	R	S	+	S	S	R	S	+	S	-	-	-	R	+	-	R	+	-	S	-	S	-	+	+	+	-	S	S	-	-	-	+	-	+	+	+	+
PT19	non-MDR	TET	S	S	-	S	S	R	S	+	S	-	-	-	S	-	-	S	-	-	S	-	S	-	-	-	+	-	S	S	-	-	-	+	-	+	+	+	+
PT20	MDR	AMP-TET-CHL-TMP-SMX-AZM	R	S	+	S	S	R	S	+	R	+	-	-	R	+	-	R	+	-	R	+	S	-	-	-	+	+	S	S	-	-	-	+	-	+	+	+	+
PT21	MDR	AMP-TET-TMP-SMX	R	S	+	S	S	R	S	+	S	-	-	-	R	+	-	R	+	-	S	-	S	-	+	+	+	-	S	S	-	-	-	+	-	+	+	+	+
PT22	MDR	AMP-TET-CHL-TMP-SMX	R	S	+	S	S	R	S	+	R	-	+	-	R	-	+	R	+	+	S	-	S	-	+	+	+	-	S	S	-	-	-	+	+	+	+	+	+
PT23	MDR	AMP-AMC-TET-CHL-TMP-SMX-AZM	R	R	+	S	S	R	S	+	R	+	-	-	R	+	-	R	+	+	R	+	S	-	-	-	+	+	S	S	-	-	-	+	-	+	+	+	+
PT24	MDR	TET-CHL-SMX	S	S	-	S	S	R	S	+	R	-	+	-	S	-	-	R	-	+	S	-	S	-	+	+	+	-	S	S	-	-	-	+	-	+	+	+	+
PT25	MDR	AMP-AMC-TET-TMP-SMX-AZM	R	R	+	S	S	R	S	+	S	-	-	-	R	+	-	R	+	-	R	+	S	-	-	-	+	+	S	S	-	-	-	+	-	+	+	+	+
PT26	MDR	TET-TMP-SMX-AZM	S	S	-	S	S	R	S	+	S	-	-	-	R	+	-	R	+	-	R	+	S	-	+	+	+	-	S	S	-	-	-	+	-	+	+	+	+
PT27	non-MDR	AMP	R	S	-	S	S	S	S	-	S	-	-	-	S	-	-	S	-	-	S	-	S	-	-	-	+	-	S	S	-	-	-	+	-	+	+	+	+
PT28	MDR	TET-TMP-SMX-AZM	S	S	-	S	S	R	S	+	S	-	-	-	R	+	-	R	+	-	R	+	S	-	+	+	+	-	S	S	-	-	-	+	-	+	+	+	+
PT29	MDR	AMP-TET-CHL-TMP-SMX	R	S	+	S	S	R	S	+	R	-	+	-	R	-	+	R	+	+	S	-	S	-	+	+	+	-	S	S	-	-	-	+	+	+	+	-	+
PT30	MDR	AMP-TET-CHL-TMP-SMX	R	S	+	S	S	R	S	+	R	-	+	-	R	-	+	R	+	+	S	-	S	-	+	+	+	-	S	S	-	-	-	+	+	+	+	-	+
PT31	MDR	AMP-TET-CHL-TMP-SMX	R	S	+	S	S	R	S	+	R	-	+	-	R	-	+	R	+	+	S	-	S	-	+	+	+	-	S	S	-	-	-	+	+	+	+	-	+
PT32	MDR	AMP-TET-CHL-TMP-SMX	R	S	+	S	S	R	S	+	R	-	+	-	R	-	+	R	+	+	S	-	S	-	+	+	+	-	S	S	-	-	-	+	+	+	+	-	+
PT33	MDR	AMP-TET-CHL-TMP-SMX	R	S	+	S	S	R	S	+	R	-	+	-	R	-	+	R	+	+	S	-	S	-	+	+	+	-	S	S	-	-	-	+	+	+	+	-	+
PT34	MDR	AMP-TET-CHL-TMP-SMX-AZM	R	S	+	S	S	R	S	+	R	+	-	-	R	+	-	R	+	-	R	+	S	-	+	+	+	-	S	S	-	-	-	+	-	+	+	+	+
PT35	MDR	AMP-TET-CHL-TMP-SMX	R	S	+	S	S	R	S	+	R	-	+	-	R	-	+	R	+	+	S	-	S	-	+	+	+	-	S	S	-	-	-	+	+	+	+	-	+
PT36	MDR	AMP-TET-TMP-SMX	R	S	+	S	S	R	S	+	S	-	-	-	R	+	-	R	+	-	S	-	S	-	+	+	+	-	S	S	-	-	-	+	-	+	+	+	+
PT37	MDR	AMP-TET-TMP-SMX-AZM	R	S	+	S	S	R	S	+	S	-	-	-	R	+	-	R	+	-	R	+	S	-	-	-	+	+	S	S	-	-	-	+	-	+	+	+	+
PT38	MDR	AMP-AMC-TET-TMP-SMX-AZM	R	R	+	S	S	R	S	+	S	-	-	-	R	+	-	R	+	-	R	+	S	-	-	-	+	+	S	S	-	-	-	+	-	+	+	+	+
PT39	MDR	AMP-AMC-TET-TMP-SMX-AZM	R	R	+	S	S	R	S	+	S	+	-	-	R	+	-	R	+	-	R	+	S	-	+	+	+	-	S	S	-	-	-	+	-	+	+	+	+
PT40	non-MDR	TET	S	S	-	S	S	R	S	+	S	-	-	-	S	-	-	S	-	-	S	-	S	-	-	-	+	-	S	S	-	-	-	+	-	+	+	+	+
PT41	MDR	AMP-TET-CHL-TMP-SMX-AZM	R	S	+	S	S	R	S	+	R	+	-	-	R	+	-	R	+	-	R	+	S	-	-	-	+	+	S	S	-	-	-	+	-	+	+	+	+
PT42	MDR	TET-CHL-TMP-SMX-AZM	S	S	-	S	S	R	S	+	R	+	-	-	R	+	-	R	+	-	R	+	S	-	+	-	+	-	S	S	-	-	-	+	-	+	+	+	+
PT43	MDR	AMP-TET-CHL-TMP-SMX-AZM	R	S	+	S	S	R	S	+	R	+	-	-	R	+	-	R	+	-	R	+	S	-	-	-	+	+	S	S	-	-	-	+	-	+	+	+	+
PT44	MDR	TMP-SMX-AZM-NAL	S	S	-	S	S	S	S	-	S	-	-	-	R	+	-	R	+	-	R	+	S	-	+	-	+	-	S	R	-	+	+	+	-	+	+	+	+
PT45	MDR	TMP-SMX-AZM	S	S	-	S	S	S	S	-	S	-	-	-	R	+	-	R	+	-	R	+	S	-	+	-	+	-	S	S	-	-	+	+	-	+	+	+	+
PT46	MDR	TET-TMP-SMX-AZM	S	S	-	S	S	R	S	+	S	-	-	-	R	+	-	R	+	-	R	+	S	-	+	+	+	-	S	S	-	-	-	+	-	+	+	+	+
PT47	MDR	AMP-AMC-TET-CHL-TMP-SMX-AZM	R	R	+	S	S	R	S	+	R	+	-	-	R	+	-	R	+	-	R	+	S	-	+	+	+	-	S	S	-	-	-	+	-	+	+	+	+
PT48	non-MDR	AMP-AMC-CHL	R	R	+	S	S	S	S	-	R	+	-	-	S	-	-	S	-	-	S	-	S	-	-	+	+	-	S	S	-	-	-	+	-	+	+	+	+
PT49	non-MDR	AMP-AMC-CHL	R	R	+	S	S	S	S	-	R	+	-	-	S	-	-	S	-	-	S	-	S	-	-	+	+	-	S	S	-	-	-	+	-	+	+	+	+
PT50	MDR	AMP-CHL-TMP-SMX	R	S	+	S	S	S	S	-	R	-	+	-	R	-	+	R	+	+	S	-	S	-	+	+	+	-	S	S	-	-	-	+	+	+	+	+	+
PT51	MDR	AMP-TET-TMP-SMX-AZM	R	S	+	S	S	R	S	+	S	-	-	-	R	+	-	R	+	-	R	+	S	-	-	-	+	+	S	S	-	-	-	+	-	+	+	+	+
PT52	MDR	AMP-AMC-TET-TMP-SMX-AZM	R	R	+	S	S	R	S	+	S	-	-	-	R	+	-	R	+	-	R	+	S	-	-	-	+	+	S	S	-	-	-	+	-	+	+	+	+
PT53	MDR	AMP-CHL-TMP-SMX	R	S	+	S	S	S	S	-	R	-	+	-	R	-	+	R	+	+	S	-	S	-	+	+	+	-	S	S	-	-	-	+	+	+	+	+	+
PT54	MDR	TET-TMP-SMX-AZM	S	S	-	S	S	R	S	+	S	-	-	-	R	+	-	R	+	-	R	+	S	-	-	+	+	-	S	S	-	-	-	+	-	+	+	+	+
PT55	MDR	AMP-TET-TMP-SMX-AZM	R	S	+	S	S	R	S	+	S	-	-	-	R	+	-	R	+	-	R	+	S	-	-	-	+	+	S	S	-	-	-	+	-	+	+	+	+
PT56	MDR	AMP-TET-CHL-TMP-SMX-AZM	R	S	+	S	S	R	S	+	R	+	-	-	R	+	-	R	+	-	R	+	S	-	+	+	+	-	S	S	-	-	-	+	-	+	+	+	+
PT57	MDR	AMP-TET-CHL-TMP-SMX-AZM	R	S	+	S	S	R	S	+	R	+	-	-	R	+	-	R	+	-	R	+	S	-	-	+	+	-	S	S	-	-	-	+	-	+	+	+	+
PT58	MDR	AMP-TET-CHL-TMP-SMX-AZM	R	S	+	S	S	R	S	+	R	+	-	-	R	+	-	R	+	-	R	+	S	-	+	+	+	-	S	S	-	-	-	+	-	+	+	+	+
PT59	non-MDR	AMP-AMC-TET	R	R	+	S	S	R	S	+	S	-	-	-	S	-	-	S	-	-	S	-	S	-	-	+	+	-	S	S	-	-	-	+	-	+	+	+	+
PT60	MDR	AMP-AMC-TET-CHL-PEF-NAL	R	R	+	S	S	R	S	+	R	-	-	+	S	-	-	S	-	-	S	-	S	-	-	-	+	-	R	R	+	-	-	+	-	+	+	+	+

AMP - Ampicillin; AMC - Amoxicillin-clavulanic acid; MEM - Meropenem; TET - Tetracycline; CHL - Chloramphenicol; TGC - Tigecycline; TMP - Trimethoprim; SMX - Sulfamethoxazole; AZM - Azithromycin; GEN - Gentamicin; PEF - Pefloxacin; NAL - Nalidixic acid; R -Resistant; S - Susceptible; (+) - Present; (-) - Absent; MDR - Multidrug resistant; *CEF - Cephalosporins refer to cefoxitin, cefotaxime, ceftazidime, ceftriaxone and cefepime. Antimicrobial resistance genes were detected using ResFinder and only positive results are presented.

Supplementary Table S2 - List of publicly available *Salmonella enterica* serovar Rissen genomes used in the present study. All read datasets were downloaded from the European Nucleotide Archive.

Strain ID	Sample source	Year	Location	ENA Accession #
BCW_2764	Human	2002	Denmark	SAMN02368641
CRJJGF_00114	Food	2004	United States	SAMN02908621
BCW_2766	Unknown	2004	Thailand	SAMN02368643
BCW_2765	Livestock	2004	Denmark	SAMN02368642
FDA00001049	Environment	2006	Turkey	SAMN02844434
FDA00001046	Environment	2006	Vietnam	SAMN02844431
FDA00004496	Animal feed	2008	United States	SAMN02918550
IEH_NGS_SAL_00172	Environment	2009	United States	SAMN02265259
FDA00002917	Environment	2009	United States	SAMN02846301
FDA00002912	Environment	2009	United States	SAMN02846296
FDA00002855	Food	2009	United States	SAMN02846239
FDA00002853	Environment	2009	United States	SAMN02846237
FDA00002914	Environment	2009	United States	SAMN02846298
FDA00002915	Environment	2009	United States	SAMN02846299
FDA00002913	Environment	2009	United States	SAMN02846297
FDA00002854	Food	2009	United States	SAMN02846238
FDA00002916	Environment	2009	United States	SAMN02846300
FDA00002850	Environment	2009	United States	SAMN02846234
FDA00002849	Environment	2009	United States	SAMN02846233
FDA00002481	Environment	2009	United States	SAMN02845865
FDA00002848	Environment	2009	United States	SAMN02846232
FDA00002852	Environment	2009	United States	SAMN02846236
FDA00002851	Environment	2009	United States	SAMN02846235
FDA00002939	Food	2009	United States	SAMN02846323
FDA00002788	Food	2009	United States	SAMN02846172
FDA00002938	Food	2009	United States	SAMN02846322
CFSAN031935	Environment	2009	United States	SAMN03577468
CFSAN031930	Environment	2009	United States	SAMN03577463
CFSAN031937	Environment	2009	United States	SAMN03577470
32309.31	Environment	2009	United States	SAMN10253329
FDA00003115	Environment	2010	United States	SAMN02846535
FDA00003545	Aquatic	2010	Vietnam	SAMN02846965
FDA00003544	Aquatic	2010	Vietnam	SAMN02846964
FNW19G96	Aquatic	2010	Vietnam	SAMN02344903
FDA00004662	Food	2010	United States	SAMN02918711
FDA00001453	Environment	2010	Dominican Republic	SAMN02844838
NC_S803	Livestock	2010	United States	SAMN07469595
NC_S812	Livestock	2010	United States	SAMN07469592
NC_S810	Livestock	2010	United States	SAMN07469594
NC_S811	Livestock	2010	United States	SAMN07469593
NC_S801	Livestock	2010	United States	SAMN07469597
NC_S802	Livestock	2010	United States	SAMN07469596
FAR0094	Food	2011	China	SAMN02345345
FSE0050	Food	2011	Vietnam	SAMN02345332
FSW0031	Environment	2011	Philippines	SAMN02345262
CFSAN030098	Food	2012	United States	SAMN03464584
H124020495	Food	2012	United Kingdom	SAMN03168707
H125180332	Food	2012	United Kingdom	SAMN03168603
FAR0091	Food	2012	Unknown	SAMN02345586

Strain ID	Sample source	Year	Location	ENA Accession #
FAR0099	Environment	2012	Mexico	SAMN02345539
PNUSAS002751	Unknown	2012	United States	SAMN06198443
CFSAN031334	Livestock	2013	Thailand	SAMN03576868
CFSAN031330	Livestock	2013	Thailand	SAMN03576864
CFSAN031335	Livestock	2013	Thailand	SAMN03576869
CFSAN031332	Livestock	2013	Thailand	SAMN03576866
CFSAN031331	Livestock	2013	Thailand	SAMN03576865
CFSAN031321	Livestock	2013	Thailand	SAMN03576855
CFSAN031320	Livestock	2013	Thailand	SAMN03576854
CFSAN031313	Livestock	2013	Thailand	SAMN03576847
CFSAN031314	Livestock	2013	Thailand	SAMN03576848
CFSAN031318	Livestock	2013	Thailand	SAMN03576852
CFSAN031312	Livestock	2013	Thailand	SAMN03576846
CFSAN031308	Livestock	2013	Thailand	SAMN03576842
CFSAN031315	Livestock	2013	Thailand	SAMN03576849
CFSAN031324	Livestock	2013	Thailand	SAMN03576858
CFSAN031309	Livestock	2013	Thailand	SAMN03576843
CFSAN031327	Livestock	2013	Thailand	SAMN03576861
CFSAN031325	Livestock	2013	Thailand	SAMN03576859
CFSAN031322	Livestock	2013	Thailand	SAMN03576856
CFSAN031316	Livestock	2013	Thailand	SAMN03576850
CFSAN031323	Livestock	2013	Thailand	SAMN03576857
CFSAN031311	Livestock	2013	Thailand	SAMN03576845
CFSAN031310	Livestock	2013	Thailand	SAMN03576844
CFSAN031319	Livestock	2013	Thailand	SAMN03576853
CFSAN031307	Livestock	2013	Thailand	SAMN03576841
CFSAN031306	Livestock	2013	Thailand	SAMN03576840
FNE0189	Food	2013	Thailand	SAMN02698353
CFSAN045275	Livestock	2013	Thailand	SAMN04431411
CFSAN045273	Livestock	2013	Thailand	SAMN04431409
CFSAN045268	Livestock	2013	Thailand	SAMN04431404
CFSAN045265	Livestock	2013	Thailand	SAMN04431401
CFSAN045263	Livestock	2013	Thailand	SAMN04431399
CFSAN045340	Livestock	2013	Thailand	SAMN04431476
CFSAN045327	Livestock	2013	Thailand	SAMN04431463
CFSAN045326	Livestock	2013	Thailand	SAMN04431462
CFSAN045324	Livestock	2013	Thailand	SAMN04431460
OH-1302304	Unknown	2013	United States	SAMN05721584
CVM N44364F	Livestock	2013	United States	SAMN06287508
CFSAN084068	Environment	2013	United States	SAMN09756362
S280	Unknown	2014	Portugal	SAMEA3476860
73107	Human	2014	United Kingdom	SAMN03479841
5965	Human	2014	United Kingdom	SAMN03479663
40781	Food	2014	United Kingdom	SAMN03478634
68669	ND	2014	United Kingdom	SAMN03477972
31626	Human	2014	United Kingdom	SAMN03476772
5839	Human	2014	United Kingdom	SAMN03476472
13420	Human	2014	United Kingdom	SAMN03473985
CFSAN045347	Livestock	2014	Thailand	SAMN04431483
CFSAN045343	Livestock	2014	Thailand	SAMN04431479
CVM N57959F	Unknown	2014	United States	SAMN04577502

Strain ID	Sample source	Year	Location	ENA Accession #
CVM N57219F	Livestock	2014	United States	SAMN04577270
NC_NCF3D0-L5	Environment	2014	United States	SAMN06322129
FDA00008934	Food	2015	Mexico	SAMN03495909
FDA00008935	Food	2015	Mexico	SAMN03495910
91210	Food	2015	United Kingdom	SAMN03480405
91281	Human	2015	United Kingdom	SAMN03480200
91246	Human	2015	United Kingdom	SAMN03480194
91209	Food	2015	United Kingdom	SAMN03479124
91211	Food	2015	United Kingdom	SAMN03478922
91208	Food	2015	United Kingdom	SAMN03478809
91212	Food	2015	United Kingdom	SAMN03476328
FDA00009504	Aquatic animal	2015	Vietnam	SAMN04148250
FSIS1502596	Livestock	2015	United States	SAMN04331735
FSIS1503283	Livestock	2015	United States	SAMN04331725
ADRDL-15-8159	Poultry	2015	United States	SAMN04240670
FSIS1605466	Livestock	2015	United States	SAMN04530405
CVM N57971	Poultry	2015	United States	SAMN04576648
182090	Human	2015	United Kingdom	SAMN04600580
185971	Human	2015	United Kingdom	SAMN04600398
129524	Human	2015	United Kingdom	SAMN04600236
CVM N57978	Poultry	2015	United States	SAMN05771731
MS150107	Human	2015	Ireland	SAMEA81466918
NC_NCF6D0-A13	Environment	2015	United States	SAMN06322119
NC_NCF6D0-A18	Environment	2015	United States	SAMN06322118
NC_NCF6D0-A4	Environment	2015	United States	SAMN06322126
NC_NCF6D0-L9	Environment	2015	United States	SAMN06322127
NC_NCF6D0-L8	Environment	2015	United States	SAMN06322128
NC_NCF6D0-A20	Environment	2015	United States	SAMN06322116
151916	Livestock	2015	Spain	SAMEA104142963
PAT-15-27861SA	Livestock	2015	Portugal	SAMEA104142939
PAT-15-19702SA	Livestock	2015	Portugal	SAMEA104142937
15Q003557	Livestock	2015	France	SAMEA104142871
ADRDL-15-2378	Livestock	2015	United States	SAMN07351348
CFSAN069222	Environment	2015	United States	SAMN07714159
2187_Se_151916	Livestock	2015	Spain	SAMEA104354224
2176_Se_PAT_15_27861SA	Livestock	2015	Portugal	SAMEA104354222
2174_Se_PAT_15_19702SA	Livestock	2015	Portugal	SAMEA104354221
34927	Livestock	2015	United States	SAMN07420494
170460	Human	2015	United Kingdom	SAMN09423139
FDA00009757	Food	2015	Jamaica	SAMN04385807
FDA00009756	Food	2015	Jamaica	SAMN04385806
191884	Human	2015	United Kingdom	SAMN09484447
195784	Human	2015	United Kingdom	SAMN10140147
FDA00010268	Food	2016	Unresolved	SAMN05232953
FDA00010267	Environment	2016	Unresolved	SAMN05232952
PNUSAS004417	Unknown	2016	United States	SAMN05877103
ADRDL-16-9998	Livestock	2016	United States	SAMN05781501
FSIS1608386	Livestock	2016	United States	SAMN06048830
CFSAN058818	Environment	2016	United States	SAMN06175263
CFSAN058816	Environment	2016	United States	SAMN06175307
FSIS1609588	Livestock	2016	United States	SAMN06229944

Strain ID	Sample source	Year	Location	ENA Accession #
FDA00011195	Animal feed	2016	United States	SAMN06214692
FDA00011193	Animal feed	2016	United States	SAMN06214694
271097	Human	2016	United Kingdom	SAMN06278620
CFSAN059804	Environment	2016	United States	SAMN06270186
ADRDL-902	Livestock	2016	United States	SAMN06330644
OH-16-23867-16	Poultry	2016	United States	SAMN07138205
OH-16-26883-1	Poultry	2016	United States	SAMN07184770
CFSAN071463	Environment	2016	United States	SAMN08017022
CVM N16S193	Poultry	2016	United States	SAMN08114066
CVM N16S274	Poultry	2016	United States	SAMN08114143
CFSAN075243	Feed	2016	United States	SAMN08395358
CFSAN071961	Environment	2016	United States	SAMN08057881
CFSAN080464	Feed	2016	United States	SAMN09071683
227073	Human	2016	United Kingdom	SAMN09403226
216348	Human	2016	United Kingdom	SAMN09403232
217006	Human	2016	United Kingdom	SAMN09610602
212747	Food	2016	United Kingdom	SAMN09634347
FSIS1710520	Poultry	2017	United States	SAMN06459939
FSIS1710555	Livestock	2017	United States	SAMN06459285
FSIS1700028	Livestock	2017	United States	SAMN06701838
FDA00011518	Food	2017	Mexico	SAMN06689639
FSIS1700448	Livestock	2017	United States	SAMN06882344
FSIS1700956	Livestock	2017	United States	SAMN06899446
FSIS1701130	Livestock	2017	United States	SAMN07141738
367310	Human	2017	United Kingdom	SAMN07155624
360374	Human	2017	United Kingdom	SAMN07155217
367265	Human	2017	United Kingdom	SAMN07180765
333422	Food	2017	United Kingdom	SAMN07180676
333421	Food	2017	United Kingdom	SAMN07180660
362108	Human	2017	United Kingdom	SAMN07180311
363492	Human	2017	United Kingdom	SAMN07180216
FSIS1701469	Livestock	2017	United States	SAMN07237724
FSIS1702121	Livestock	2017	United States	SAMN07260890
FSIS1703115	Livestock	2017	United States	SAMN07424747
FSIS1703367	Livestock	2017	United States	SAMN07501501
FSIS1703368	Livestock	2017	United States	SAMN07501502
FDA00012093	Animal feed	2017	Argentina	SAMN07510071
PNUSAS021809	Unknown	2017	United States	SAMN07561485
FSIS21720373	Livestock	2017	United States	SAMN07819022
FSIS11704588	Livestock	2017	United States	SAMN07835489
FDA00012295	Aquatic animal	2017	Vietnam	SAMN07981379
FSIS11705536	Livestock	2017	United States	SAMN08114352
CFSAN071971	Environment	2017	United States	SAMN08057833
CFSAN071970	Environment	2017	United States	SAMN08057822
FSIS1701253	Livestock	2017	United States	SAMN09098834
412138	Human	2017	United Kingdom	SAMN09298461
CFSAN081790	Feed	2017	United States	SAMN09262024
456191	Human	2017	United Kingdom	SAMN09388861
371818	Human	2017	United Kingdom	SAMN09423133
388672	Human	2017	United Kingdom	SAMN09431486
445933	Human	2017	United Kingdom	SAMN09445526

Strain ID	Sample source	Year	Location	ENA Accession #
378690	Food	2017	United Kingdom	SAMN09643791
CVM N17S1456	Livestock	2017	United States	SAMN09771059
413029	Human	2017	United Kingdom	SAMN09433805
PNUSAS032562	Environment	2018	United States	SAMN08383811
FSIS31800061	Livestock	2018	United States	SAMN08432325
ADRDL-1843	Livestock	2018	United States	SAMN08579906
FSIS11808313	Livestock	2018	United States	SAMN08767328
FSIS21821362	Poultry	2018	United States	SAMN08848643
FSIS11808806	Livestock	2018	United States	SAMN08886742
FSIS11809218	Livestock	2018	United States	SAMN08967083
529250	Human	2018	United Kingdom	SAMN09076426
FSIS21821653	Poultry	2018	United States	SAMN09225336
520599	Human	2018	United Kingdom	SAMN09388937
FSIS11810650	Livestock	2018	United States	SAMN09444133
533995	Human	2018	United Kingdom	SAMN09522091
sam	Human	2018	Ireland	SAMEA4730812
550127	Human	2018	United Kingdom	SAMN09607167
503417	Human	2018	United Kingdom	SAMN09634155
526142	Human	2018	United Kingdom	SAMN09651916
FSIS11811376	Livestock	2018	United States	SAMN09633410
570850	Human	2018	United Kingdom	SAMN09683619
FSIS11810870	Poultry	2018	United States	SAMN09533147
FSIS31800980	Poultry	2018	United States	SAMN09994491
FSIS11813906	Livestock	2018	United States	SAMN10064108
FSIS11808310	Livestock	2018	United States	SAMN08767326
06-1346	Unknown	Unknown	Unknown	SAMEA1484347
PNUSAS001935	Unknown	Unknown	United States	SAMN04893774
VNB1121-sc-2280660	Unknown	Unknown	Unknown	SAMEA3447772
2452-sc-2280574	Unknown	Unknown	Unknown	SAMEA3447686
74_H_097-sc-2280751	Unknown	Unknown	Unknown	SAMEA3447861
72_H_175-sc-2280732	Unknown	Unknown	Unknown	SAMEA3447843
71_H_195-sc-2280689	Unknown	Unknown	Unknown	SAMEA3447801
71_H_131-sc-2280683	Unknown	Unknown	Unknown	SAMEA3447795
VNSC2361-sc-2280610	Unknown	Unknown	Unknown	SAMEA3447722
VNB1504-sc-2280704	Unknown	Unknown	Unknown	SAMEA3447816
01-0479	Unknown	Unknown	Unknown	SAMN03264955
00-0084	Unknown	Unknown	Unknown	SAMN03264954
2012K-0157	Unknown	Unknown	Unknown	SAMN03264953
BCW_2864	Unknown	Unknown	Unknown	SAMN02368730
PNUSAS002711	Unknown	Unknown	United States	SAMN05603672
PNUSAS004670	Unknown	Unknown	United States	SAMN05919885
S02948-14	Unknown	Unknown	Unknown	SAMEA4064117
S02707-14	Unknown	Unknown	Unknown	SAMEA4064114
PNUSAS006373	Unknown	Unknown	United States	SAMN06213966
SAMEA4412641	Unknown	Unknown	Unknown	SAMEA4412641
PNUSAS007242	Unknown	Unknown	United States	SAMN06220355
PNUSAS007916	Unknown	Unknown	United States	SAMN06310818
PNUSAS009904	Unknown	Unknown	United States	SAMN06625047
PNUSAS014086	Unknown	Unknown	United States	SAMN07161029
PNUSAS023317	Unknown	Unknown	United States	SAMN07806985
PNUSAS025277	Unknown	Unknown	United States	SAMN07949738

Strain ID	Sample source	Year	Location	ENA Accession #
PNUSAS027824	Unknown	Unknown	United States	SAMN08016445
PNUSAS030901	Unknown	Unknown	United States	SAMN08240237
PNUSAS032846	Unknown	Unknown	United States	SAMN08432357
PNUSAS029879	Unknown	Unknown	United States	SAMN08470840
PNUSAS035109	Unknown	Unknown	United States	SAMN08616364
PNUSAS036743	Unknown	Unknown	United States	SAMN08823502
PNUSAS037800	Unknown	Unknown	United States	SAMN08865617
PNUSAS037136	Unknown	Unknown	United States	SAMN08865508
PNUSAS037130	Unknown	Unknown	United States	SAMN08865514
PNUSAS037097	Unknown	Unknown	United States	SAMN08922760
PNUSAS042247	Unknown	Unknown	United States	SAMN09381922
PNUSAS042313	Unknown	Unknown	United States	SAMN09501102
PNUSAS043909	Unknown	Unknown	United States	SAMN09534158
PNUSAS044558	Unknown	Unknown	United States	SAMN09636247
PNUSAS045202	Unknown	Unknown	United States	SAMN09650791
14ARS_VSM0382	Unknown	Unknown	Unknown	SAMEA104162236
14ARS_VSM0381	Unknown	Unknown	Unknown	SAMEA104162235
